# Enabling personalized perioperative risk prediction by using a machine-learning model based on preoperative data

**DOI:** 10.1038/s41598-023-33981-8

**Published:** 2023-05-02

**Authors:** Martin Graeßner, Bettina Jungwirth, Elke Frank, Stefan Josef Schaller, Eberhard Kochs, Kurt Ulm, Manfred Blobner, Bernhard Ulm, Armin Horst Podtschaske, Simone Maria Kagerbauer

**Affiliations:** 1 Department of Anaesthesiology and Intensive Care Medicine, School of Medicine, Technical University of Munich, Munich, Germany; 2 Department of Anaesthesiology and Intensive Care Medicine, School of Medicine, University Hospital Ulm, Albert-Einstein-Allee 23, 89081 Ulm, Germany; 3 Commercial department, Klinikum rechts der isar, Technical University of Munich, Munich, Germany; 4 Department of Anaesthesiology and Operative Intensive Care Medicine (CVK, CCM), Charité-Universitätsmedizin Berlin, Corporate Member of Freie Universität Berlin, Humboldt-Universität zu Berlin, and Berlin Institute of Health, Berlin, Germany; 5 Department of Medical Statistics and Epidemiology, School of Medicine, Technical University of Munich, Munich, Germany

**Keywords:** Medical research, Outcomes research, Risk factors

## Abstract

Preoperative risk assessment is essential for shared decision-making and adequate perioperative care. Common scores provide limited predictive quality and lack personalized information. The aim of this study was to create an interpretable machine-learning-based model to assess the patient’s individual risk of postoperative mortality based on preoperative data to allow analysis of personal risk factors. After ethical approval, a model for prediction of postoperative in-hospital mortality based on preoperative data of 66,846 patients undergoing elective non-cardiac surgery between June 2014 and March 2020 was created with extreme gradient boosting. Model performance and the most relevant parameters were shown using receiver operating characteristic (ROC−) and precision-recall (PR-) curves and importance plots. Individual risks of index patients were presented in waterfall diagrams. The model included 201 features and showed good predictive abilities with an area under receiver operating characteristic (AUROC) curve of 0.95 and an area under precision-recall curve (AUPRC) of 0.109. The feature with the highest information gain was the preoperative order for red packed cell concentrates followed by age and c-reactive protein. Individual risk factors could be identified on patient level. We created a highly accurate and interpretable machine learning model to preoperatively predict the risk of postoperative in-hospital mortality. The algorithm can be used to identify factors susceptible to preoperative optimization measures and to identify risk factors influencing individual patient risk.

## Introduction

Postoperative all-cause mortality is approximately 0.5% for elective procedures; however, this percentage varies by procedure and urgency status^1^. Accurate knowledge of individual patient risk is essential to raise awareness for early recognition of postoperative complications and adequate planning of intraoperative management and postoperative care. Furthermore, from an ethical and legal point of view, the patient has the right to know his or her risk of the planned procedure to enable shared decision making with physician and patient as equal partners^2,3^. To meet these needs, current guidelines recommend the application of numerous scores for risk assessment. One of the oldest ones is the American Society of Anaesthesiologists Physical Status (ASA-PS), in use since 1941 and revised several times in the past^4^. More recent scores, for example the surgical Apgar score or the POSSUM (Physiological and Operative Severity Score for the enUmeration of Mortality and morbidity) require intraoperative variables for calculation and are therefore not suitable for preoperative risk evaluation^5^. In 2016, Le Manach and co-workers developed the POSPOM (PreOperative Score to predict PostOperative Mortality) consisting of preoperative factors like age, comorbidities and type of surgery^6^. All these scores predicting overall mortality risk may perform well on a population level, but they allow only limited personalized statements about the individual patient which, however, is necessary to achieve shared decision-making.

Recently, the Covid-19 pandemic gave a boost to the development of machine learning algorithms for prediction and triage of ICU patients^7^. More and more, such algorithms are also being developed in the field of perioperative medicine, showing promising results^8,9^. Due to the increasing use and efficiency of big data analyses and artificial intelligence in healthcare, machine learning algorithms have turned out to be superior to traditional scores in prediction accuracy^8^.

Machine learning models are complex mathematical constructs that often cannot even be fully understood by the person who has programmed them, they form so-called “black boxes”. Medical ethicists therefore call for transparent models whose decision a physician can also understand^10^. However, these are not so easy to implement, since it is postulated that a model loses its predictive accuracy with increasing explicability^11^. Nevertheless, with careful model design there is hope that transparent, interpretable algorithms can not only help to identify patients at risk, but also may reveal factors which can be optimized preoperatively.

Consequently, the aim of our study was to create a machine learning algorithm to accurately predict postoperative in-hospital mortality based on preoperative factors. A further objective was to make the model comprehensible for the physician. Interpretability shall be reached by creating personalized risk profiles and carrying out thought experiments on changing identified risk factors in the model to determine their influence on patient outcome.

## Methods

The study was designed in accordance with the TRIPOD statement concerning multivariable prediction models for individual diagnosis^12^.

### Participants

**Figure 1:**
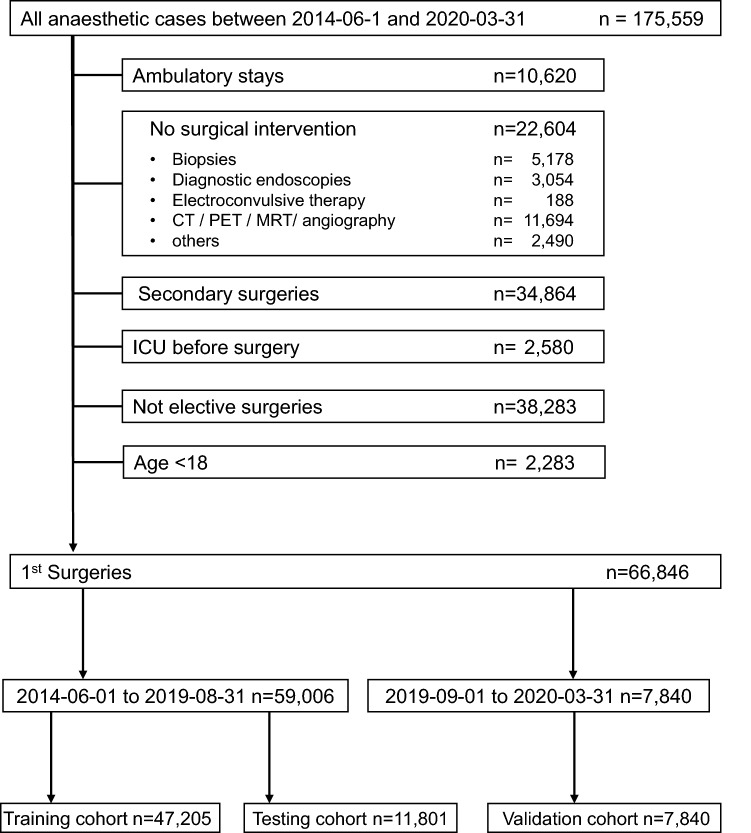
STROBE diagram. *CT* computed tomography, *PET* positron emission tomography, *MRT* magnetic resonance tomography, *ICU* intensive care unit.

After approval (253/19 S-SR of 11-Jun-2019) by the Ethics Committee of the Medical Faculty of the Technical University of Munich (Ethikkommission der Technischen Universität München, https://www.ek-med-muenchen.de/) and registration in ClinicalTrials.gov (NCT04092933), the study was conducted at the university hospital of the Technical University of Munich. Informed consent was waived from all subjects or their legal guardians according to German regulations due to retrospective analysis of routine data. The study was performed in accordance with ethical guidelines, recommendations of the German Ethics Council and legal regulations. In accordance with legal data protection requirements, all identifying information had been removed from the patient records used. Retrospective analysis included data of adult patients during each elective first non-cardiac surgery within a hospital stay between June 2014 and March 2020. Follow-up surgery in patients who underwent multiple surgical procedures as well as patients being admitted to ICU prior to the first surgery were excluded. Outpatient surgeries and minor cases like patients undergoing diagnostic procedures or electroconvulsive therapy were also excluded (Fig. 1). In case that a patient was admitted to hospital more than once during the 6-year observation period, these cases were considered separately if they were assigned different case numbers in the hospital information system.

### Source of data

All data were derived from three different sources: the hospital information system, the laboratory information system, and the patient data management system. The hospital information system and the patient data management system work largely independently of each other and are only equipped with interfaces for exchanging the core data such as case number and patient ID. Therefore, these data sources had to be queried separately. All data of the laboratory information system is fully integrated into the clinic information system via a technical interface.

### Outcome

Primary endpoint was in-hospital mortality which is a commonly used quality parameter in many countries. The general definition of this endpoint is “death in hospital during the index admission”^13^. Parameters with the greatest overall contribution to the predictive power of the model were identified. Furthermore, the model was used to compute individual risk profiles of exemplary patients and to visualize alterations in risk as parameters change.

### Features, pre-processing, and missing data

Our model included all preoperatively available data derived from the various digital documentation systems of the hospital. Laboratory values, blood orders, surgical procedure (OPS) codes, and in-hospital movements were already in tabular and structured form.

The patients’ medical history was given in free text. To extract relevant information, we first searched for medical terms excluding stop words and phrases. The resulting list contained the medical terms with the highest frequency. In the next step we searched for these terms and in addition looked for negations. We created each medical term as a feature with the categories "yes", "no" and "not available".

Current patient medication was also given as free text including spelling and typing errors. Therefore, we developed a workflow which extracted the drug names, conducted a spellcheck, and assigned the drug to its Anatomical Therapeutic Chemical (ATC) Code. The first four digits of the ATC code were used to group the drugs into substance classes, which were then used in the model.

For the laboratory tests included, a time window of two weeks before the respective surgery was determined. From this period, the laboratory value closest to the surgery date was selected.

Missing values were not imputed. A dichotomous feature of each variable included information about its availability. Distribution and missingness of the most important features in each cohort are shown in Table A1 of the appendix.

### Sample size

The dataset which was obtained between June 2014 until August 2019 was used as training and test cohort by a stratified 4:1 split. The test cohort was used to tune the hyperparameters and to avoid overfitting. After completing the training and testing, additional data collected between September 2019 and March 2020 was used to allow validation of the model, i.e., to see how well the model performs on unseen data. This resulted in a training cohort of 47,205, a testing cohort of 11,801 and a validation cohort of 7,840 hospital stays.

### Model development

Prediction models were developed using extreme gradient boosting (XGBoost)^14^ with the tuning parameters “learning rate”, “minimum loss reduction”, “maximum depth of each tree”, “fraction of features”, “fraction of training samples”, “scale of positive weights”, and “minimum sum of instance weight” (for details see Chen and co-workers^15^). The results of the XGBoost models are highly depending on these parameters. Parameter optimization is both time and computationally intensive. We used Bayesian hyperparameter search for tuning parameters for maximum area under the precision recall curve (AUPRC) and used threefold cross validation on the training set due to the size of the data set^16^. Hyperparameter settings were as follows: eta (learning rate) = 0.0549; gamma (minimum loss reduction) = 1.69; max_depth (maximum depth of each tree) = 2; min_child_weight (minimum sum of instance weight) = 6; subsample (fraction of training samples) = 0.801; colsample_bytree (fraction of features) = 0.918; scale_pos_weight (scale of positive weights) = 4.07. The hyperparameter “scale of positive weights” is necessary in this case to correct for a highly imbalanced dataset, as mortality is about 0.5% in our patient cohort.

The starting point for model development was over 12,000 parameters, including more than 9300 OPS codes. Due to their infrequent occurrence, a large number of them was not included in the final model, leaving 201 parameters, a list of which is provided in the appendix (Table A2).

The model was calibrated using isotonic regression. Calibration metrics and plots are shown in Table A3 and Fig. A4 of the appendix.

### Statistical analysis and model interpretation

Analysis was performed using R (version 4.1.2, R Foundation for Statistical Computing; Vienna, Austria). Predictive quality of the model is demonstrated by its area under receiver operating characteristic (AUROC) and area under precision-recall curve (AUPRC) [95% confidence interval]. The receiver operating characteristic (ROC) plot shows the trade-off between specificity and sensitivity, and the AUROC is the most widely used measure to evaluate a classifier’s performance. Additionally, we show precision-recall-curves (PRC) to depict the fraction of true positives among the whole number of positives with a baseline that depends on class distribution^17^. We calculated AUROC as well as AUPRC on the validation set. 95% confidence intervals of ROC and PR curve were calculated by means of the ci.auc function using 2000 stratified bootstrap samples.

The variables that contribute most to the prediction are visualized in an importance plot. These plots depict the gain, which shows the relative contribution of each feature to the model by calculating the share for every single tree using the leave-one-covariate-out method^18^.

Partial dependence plots show the change in risk with increasing or decreasing variable values.

Individual risk profiles of exemplary patients are presented by means of waterfall plots, which show the impact of the individual variables on the overall prediction of the respective patient. The effect of changing a factor, all other things being unaffected, was determined and graphically represented by means of ceteris-paribus-plots. These plots were created by gradually changing a specific parameter and calculating the resulting risk. The value of the parameter was plotted on the x-axis, and the respective risk was then plotted on the y-axis. Consequently, these plots show us how the prediction would change if we modify just one risk factor leaving the others equal.

### Consent statement

Informed consent was waived by the Ethics Committee of the Medical Faculty of the Technical University of Munich (Ethikkommission der Technischen Universität München, https://www.ek-med-muenchen.de/) due to the retrospective nature of the study (253/19 S-SR of 11-Jun-2019).

## Results

### Participants

Excluding underage patients, secondary surgeries, ICU patients, diagnostic and emergency procedures, 66,846 surgeries from a total of 175,559 remained. Here, 59,006 interventions that took place between June 2014 and August 2019 served as the training and testing dataset, and interventions between September 2019 and March 2020 formed the dataset for external validation (Fig. 1).

Over all cohorts, median age was 58 years [interquartile range (IQR) 43–71] with most patients categorized in ASA class II (51.9%), 45% were female. Overall mortality was 0.5%. Surgical procedure codes (German OPS) and procedural data were available for all patients. Feature distributions of training, testing and validation cohort are given in the appendix (Table A1).

### Model characteristics

**Figure 2:**
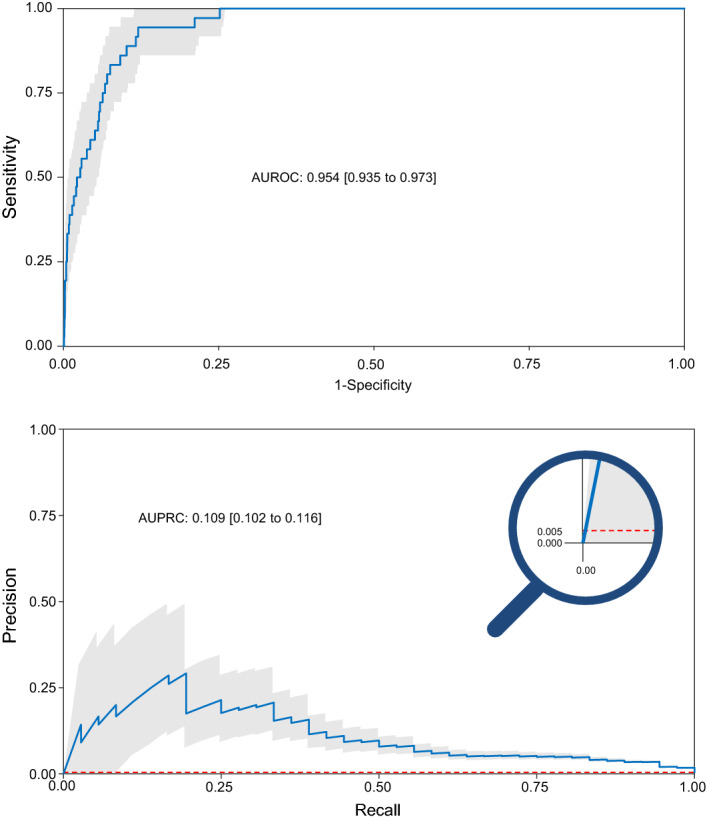
Receiver-operating characteristic (ROC) and precision-recall (PR) curves. ROC curves as depicted for our model on the upper side look the same for different classifiers, regardless of the basic probability, and are often used to assess the predictive quality of a model. An area under the curve of 1.0 would mean a perfect, an area under the curve of 0.5 a random classifier. In the PRC as shown below, the baseline is determined by the proportion of positives and negatives. As overall mortality is 0.5% in our patient cohort, the baseline in our model is quite low (0.005). This is depicted by the red dashed line which corresponds to the performance of a random classifier. The area under the curve here shows us how to evaluate a positive result of the classifier given the basic probability^17^. The shaded area represents the 95% confidence interval.

**Figure 3:**
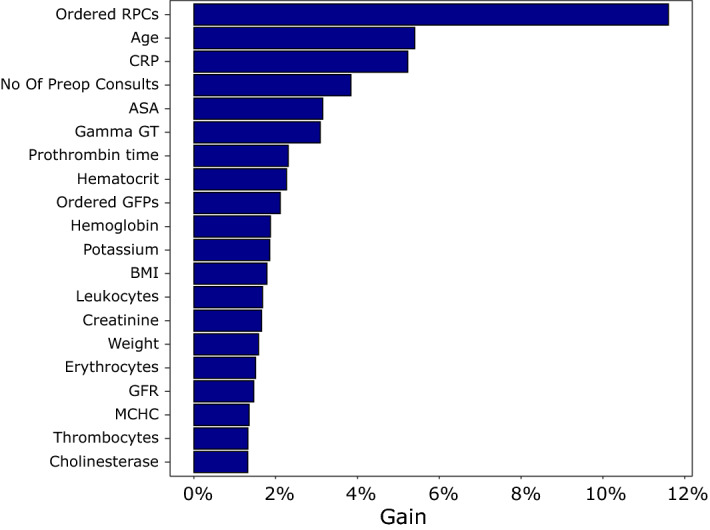
Importance plot. This figure shows the twenty most important factors and their contribution to model prediction. *RPCs* red packed cells, *CRP* c-reactive protein, *ASA* American Society of Anaesthesiologists Physical Score, *Gamma GT* gamma-glutamyl-transferase, *GFPs* fresh frozen plasma, *BMI* body-mass index, *GFR* glomerular filtration rate, *MCHC* mean corpuscular haemoglobin.

The model shows good predictive ability with an AUROC of 0.954 [IQR 0.935–0.973] and a AUPRC of 0.109 [IQR 0.102–0.116] (Fig. 2) calculated on the validation set. The most important factors contributing to the model are the number of ordered red packed cells, age and c-reactive protein, number of preoperatively requested consults and ASA-PS (Fig. 3). The top twenty variables were all numerical. Information derived from free text fields like medication or facts from the patient history contributed less to the model’s predictive ability. An overview of all features used in the model is given in the appendix (Table A2). Positive and negative predictive values, F1 scores and sensitivity depending on the probabilities calculated by the model are shown in Fig. A5 of the appendix.

### Interpretability on model level

**Figure 4:**
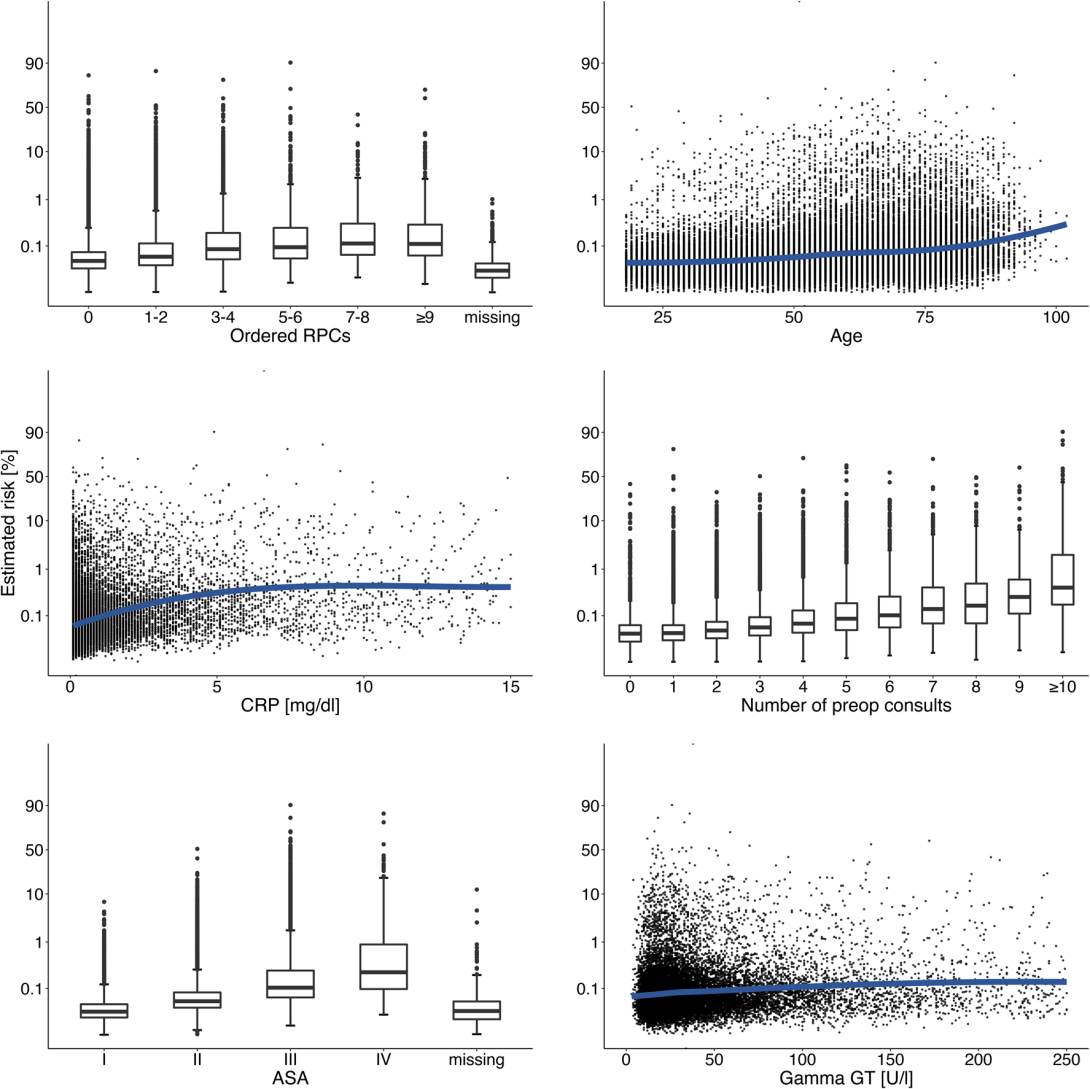
Partial dependence plots. This figure shows the top six factors and their influence on patient risk. The y-axis represents mortality risk in a logit-scale. Factors with discrete values (ordered RPC’s, number of preop consults, ASA score) are depicted by boxplots with median and interquartile range representing estimated patient risk. Regarding age in yearly intervals and the concentrations of CRP and Gamma GT as continuous values, mortality risk rises with increasing parameter values. *RPCs* red packed cells, *CRP* c-reactive protein, *ASA* American Society of Anaesthesiologists Physical Score, *Gamma GT* gamma-glutamyl-transferase.

Partial dependence plots show the change in risk with increasing or decreasing variable values. The mortality risk rises with increasing number of ordered packed red cells (PRC’s), age, c-reactive protein, number of preoperative consults, ASA score and Gamma-GT (Fig. 4).

### Interpretation on patient level

To illustrate personalised prediction, we used the model to calculate the risk of two exemplary patients and plotted the individual risk factors and their contribution to the single patient's overall risk with waterfall plots. The two patients were selected because they cover prehabilitation and patient blood management, two important topics in preoperative evaluation. Furthermore, in a model calculation, it was shown how the patients’ risk would behave if defined factors were changed. The result is presented in so-called ceteris-paribus-plots.

**Figure 5:**
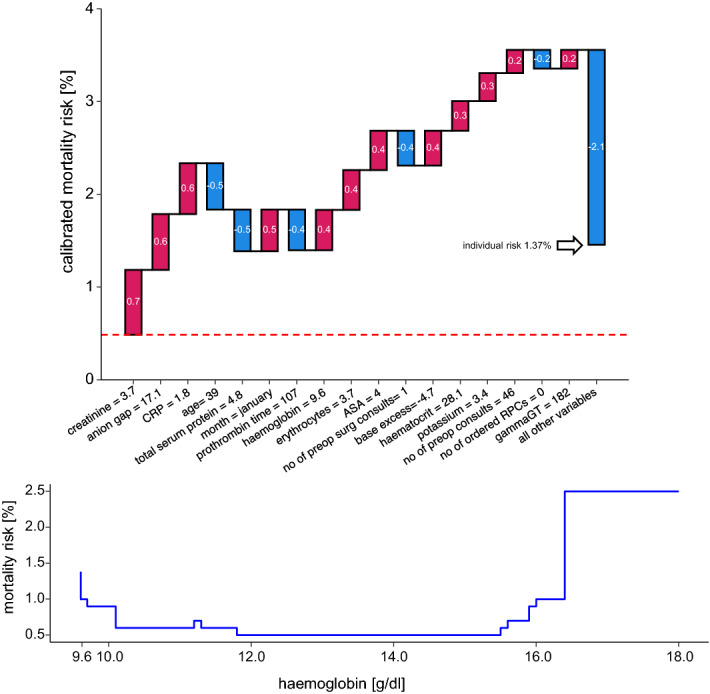
Example patient 1. Waterfall plots depict explanations for individual predictions. Their input is a vector of variables which account for the prediction of a single patient. The bottom of a waterfall plot starts at the baseline risk of our overall cohort (here: 0.5%). Red bars represent variables that increase risk, blue bars represent those that decrease risk. The patient’s mortality risk is shown on the y-axis. The baseline changes with the contribution of each value to overall risk and ends at the individual risk estimation of the respective patient. The waterfall plot in this figure depicts the risk profile of a 39-year-old man with an overall in-hospital mortality risk of 1.37%. Numerical variables contribute most to model prediction. Most laboratory values may serve as surrogate parameters for organ dysfunction. A low haemoglobin value of 9.6 is conspicuous, which can possibly be optimized by adequate preoperative therapy. The plot below depicts the change of the risk profile by manipulating the haemoglobin value. In these so-called ceteris paribus plots, it is assumed that only one variable is changed and all others remain the same. Of course, the haematocrit also changes with the haemoglobin. In this simulation, haematocrit was estimated to be three times the value of the haemoglobin and adjusted accordingly.

Patient 1 is a 39-year-old male, ASA IV, with an overall risk for in-hospital mortality of 1.37%. Looking for potentially modifiable risk factors, we find preoperatively measured haemoglobin being as low as 9.6 mg/dl. If we now increased the haemoglobin value preoperatively through targeted anaemia therapy, we would have to face the difficulty that this will additionally change the haematocrit which also contributes to the model’s prediction as a factor. In order to do justice to this connection, we have assumed that the haematocrit is threefold times the haemoglobin value according to a common estimate^19^. The plot below then shows us that if we managed to increase the haemoglobin value by 0.5 points, the risk would be reduced from 1.37 to 0.6% (Fig. 5).

**Figure 6:**
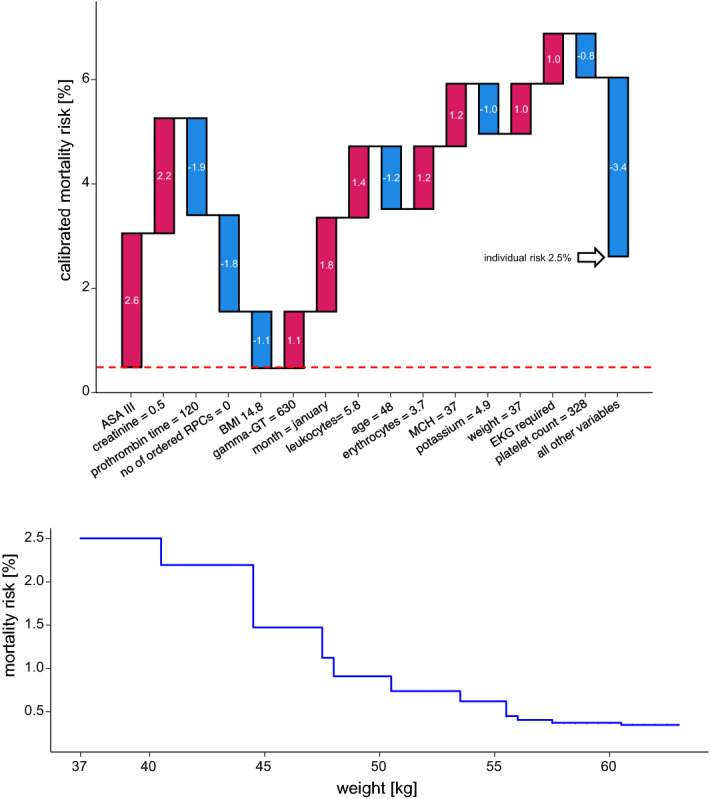
Example patient 2. This cachectic patient is a 48-year-old female with a mortality risk of 2.5%. The waterfall plot shows the patient’s individual risk profile. One probably modifiable risk factor is weight. Weight gain would also lead to an increase in body-mass-index; therefore, the BMI was adjusted accordingly. The second plot shows how the risk decreases with increasing weight.

The second example patient is a 48-year-old woman, ASA III, with a baseline risk of 2.5%. Looking for potentially modifiable risk factors we find that the patient is underweight with a body-mass-index (BMI) of 14.8. Again, we have to take into account that in the model two interrelated factors exert an influence, BMI and weight. Since this is a linear relationship described by a known formula, it is possible here to calculate the influence of a change in weight with a consecutive change in BMI as an example. The resulting plot shows that we would need to raise the weight of this patient by about eight kilograms preoperatively to reduce mortality by 1% (Fig. 6).

## Discussion

Using extreme gradient boosting, a machine learning technique, we created a model which was able to predict postoperative in-hospital mortality for an individual patient with high accuracy. The most important variables were number of preoperatively ordered red packed cells, c-reactive protein and age. Tabular data contributed most to the models’ predictive value whereas unstructured data like free-text had less impact on model performance. Individual risk factors and the influence of changes in individual factors were calculated and displayed graphically making the model interpretable. In clinical routine, the model could be useful for physicians and patients to support informed decision-making.

Regarding the most important variables in our model, the number of red packed cells provided depends primarily on the type of procedure. Pre-existing conditions of the patient may also play a role. Most hospitals have standards that determine how many units of blood are provided prior to surgery and are based on valid guidelines and internal hospital transfusion benchmarks. Therefore, this factor does not reflect the purely subjective assessment of the physician.

The relationship between age and mortality risk is undisputed. Age is included as a factor in many conventional scores, including the POSPOM and Charlson Comorbidity Index mentioned earlier^6,20^. High values of c-reactive protein may indicate infection and are associated with a poor level of cardiorespiratory fitness. Higher pre-operative c-reactive protein concentrations have been shown to be associated with postoperative complications^21^. Thus, these factors appear relevant to postoperative outcome, supporting the plausibility of our model.

Preoperative risk assessment is essential to identify patients with an increased risk of morbidity and mortality and to develop perioperative strategies to minimize those risks^2^. In addition, knowing the risk helps to adequately inform and involve the patient in decisions concerning the planned surgery. Therefore, current guidelines recommend to assess the patient’s risk for perioperative complications by using various scores^2^. The most common one, ASA-PS, however, showed only poor predictive abilities for postoperative mortality with a recently reported AUROC of about 0.63^22^ and therefore seemed not suitable for reliable mortality prediction. Recently, more complex scores are preferred, for example the POSPOM or CCI (Charlson Comorbidity Index)^6,20^. However, predictive ability of these scores does not really exceed that of the ASA-PS, as AUROCs are reported of 0.64 for the CCI and 0.65 for the modified frailty index^23^. Although the original report of the POSPOM score by LeManach et al. seemed to be able to keep up with the ASA showing an AUROC of 0.944, it has to be taken into account that a validation of the POSPOM in the respective country including a matching of surgical codes has to be performed. The German validation of the POSPOM reported by Layer et al. shows only an AUROC of 0.771^24^.

The potential of applying machine learning methods in perioperative medicine was confirmed by a recent systematic review in which it was noted that many models were able to reach an AUROC of more than 0.9 and therefore outperform most conventional scores^25^. The review further confirmed that random forests and gradient boosting were most frequently used and showed best model performance^8^. The results of our study were consistent with these results as, using XGBoost, we achieve an AUROC of 0.95 and an acceptable precision-recall trade-off^26^.

However, to effectively improve patient outcome and adapt the perioperative approach to the patient, more than just knowledge of the risk is necessary. Simplified conventional scores only provide population-level predictions. Our machine-learning model however opens the gates to personalized medicine in the field of anaesthesia as it allows to identify modifiable risk factors in every single patient.

To make such a complex model interpretable to clinicians, we can calculate the impact of every single parameter on the predictive power of the model. Furthermore, we can calculate the change of risk when modifying a single factor under the condition that all others remain the same. This is, however, a very theoretical approach, as many factors interact with each other, and we don’t always know which factors are interconnected in which way. Similar to conventional retrospective analyses, we have to acknowledge the effect of unidentified confounders and multicollinearity. While not critical for the goodness of the model’s fit, multicollinearity most likely dilutes the impact of the critical factors^27^. Nevertheless, in selected cases, the model can be quite useful to illustrate the effect of preoperatively initiated optimization measures. To do this, however, it is necessary to know the dependent factors and to be able to model their interrelationships mathematically. We here give an example by illustrating two cases that concern two current topics: patient blood management and prehabilitation, the attempt to preoperatively improve the functional capacity of a patient.

In the first example, we see a significant reduction in perioperative mortality risk with rising haemoglobin levels. Current guidelines recommend preoperative anaemia assessment and therapy with a threshold that lies at a haemoglobin level < 13 g/dL in males and < 12 g/dL in menstruating females^28^. What is remarkable in our exemplary patient is that, after having reached a minimum, mortality risk rises again with increasing Hb, but the optimum lies within the threshold defined by the World Health Organization (WHO).

Our second example fits the increasingly important issue of prehabilitation especially in older and frail patients to build up their decreased reserves. Recently, a meta-analysis showed that preoperative optimization measures can reduce postoperative morbidity. Unfortunately, uniform protocols and procedures do not yet exist, and the influence of prehabilitation measures on postoperative outcomes is not yet known. Mostly multimodal concepts are pursued, as it is not yet clear which patient benefits from which preoperative intervention^29,30^. Here, we demonstrate the effect of weight gain in a cachectic patient, from which one might conclude that this patient would benefit from preoperative nutritional therapy. However, with an overall rather low mortality risk, the effect of weight gain is not too pronounced.

In addition to the aforementioned implications of multicollinearity, we have to face some more challenges and limitations: we here provide only a single centre study, however with a considerable number of patients. One national speciality in our study are surgical procedures classified by OPS (German: “Operationen und Prozedurenschlüssel”, operations and procedure codes). The German OPS describes surgical procedures at a very fine granular level. The majority of these codes did not appear in the model due to low frequencies. However, since this classification is primarily used for billing purposes, grouping is not possible without loss of information which is why we refrained from aggregating the codes. In addition, we deliberately left some duplicates of variables, for example glomerular filtration rate (GFR) calculated according to two different formulas, as these give different values in different patient groups. Another limitation of our model is the lack of intraoperative information, which can still decisively change the mortality risk compared to the preoperative setting. It is obvious that information such as the duration of surgery and intraoperative blood loss or the occurrence of adverse events can have critical influence on the postoperative course^31^. However, for assessing and counselling a patient during the pre-anaesthesia visit before an elective surgical procedure, our model provides reliable information.

Clinical documentation is often incomplete, which leads us to have missing data on individual variables. We accounted for this problem by adding for each variable an additional dichotomous feature including information about its availability. Interestingly, in the final model, only two of these dichotomous features remained, namely “bilirubin available” and “main diagnosis available” (see Table A2 of the appendix).

Another crucial factor is that the quality of preoperative data collected on a routine basis is often insufficient. This fact is reflected in our model, as numerical and tabular data contribute most to the prediction. Data derived from free text was not represented among the top 200 variables. Therefore, many of the factors that appear in the model, especially the laboratory values, are simply surrogate parameters for organic diseases that are better described in findings or physicians' reports. However, important information about the patient's preoperative condition is usually still collected as free text in clinical routine. This unstructured information can only be inadequately processed by such a complex model. There are now two ways to remedy this fact. On the one hand, natural language processing methods could be refined and integrated into the model. There is evidence that the inclusion of such algorithms in models improves prediction quality^32^. However, a very heterogeneous set of algorithms is available, some of which have not yet been externally validated^33^. Another option is to avoid the extensive use of free text and to force the user to structured and complete inputs by the user interface. This would entail a major redesign of most clinical documentation tools. In times when interoperability between different medical documentation systems is becoming increasingly important, structured information capture in a uniform document architecture will become an important prerequisite. There is hope that uniform nomenclatures and syntactic and semantic standards will make scientific evaluation as well as the use of the data or the creation of prediction models much easier in the future.

In conclusion, our study demonstrates that it is feasible to create a machine-learning model to predict the risk of postoperative in-hospital mortality with good accuracy outperforming traditional scores. The model can be used to determine risk factors on a personalized level and therefore presents a suitable basis for informed consent in high-risk patients. Further, we made the model interpretable by calculating the impact of a change in modifiable risk factors for selected cases. Thus, our model is suitable to identify personalized risk of mortality and to evaluate the effect of modifying risk factors in future studies.

## Supplementary Information

Supplementary Information.

## Data Availability

Due to legal requirements, we are not allowed to store data, although it is de-identified, in a publicly accessible repository. To gain access, proposals should be directed to the corresponding author. Requestors will need to sign a data access agreement.
