# Supplementary material for: Enabling personalized perioperative risk prediction by using a machine-learning model based on preoperative data
Source: Sci Rep. 2023 May 2;13:7128. doi: 10.1038/s41598-023-33981-8 (PMC10153050; doi:10.1038/s41598-023-33981-8)
Supplement: Supplementary file 1 — Supplementary Information. [file 41598_2023_33981_MOESM1_ESM.docx]

# Appendix

## Supplementary materials

### Table A1: Properties of the training, testing and validation cohort:

| **Parameter** | **Training, N = 48,417** | **Testing, N = 12,075** | **Validating, N = 11,026** |
| --- | --- | --- | --- |
| general |  |  |  |
| no of patients died | 242 (0.5%) | 69 (0.6%) | 67 (0.6%) |
| sex female | 21,824 (45%) | 5,372 (44%) | 4,882 (44%) |
| age | 58 (42, 71) | 58 (43, 71) | 59 (43, 71) |
| body-mass-index | 25.5 (22.8, 28.7) | 25.5 (22.8, 28.7) | 25.4 (22.8, 28.8) |
| unknown | 4.8% | 4.7% | 0.9% |
| number of preop consults | 2 (1, 3) | 2 (1, 3) | 3 (2, 4) |
| surgery outside regular service | 531 (1.1%) | 145 (1.2%) | 58 (0.5%) |
| ASA |  |  |  |
| I | 12,704 (27%) | 3,068 (26%) | 2,458 (22%) |
| II | 25,226 (53%) | 6,335 (53%) | 5,565 (51%) |
| III | 9,376 (20%) | 2,374 (20%) | 2,852 (26%) |
| IV | 377 (0.8%) | 125 (1.1%) | 136 (1.2%) |
| ASA missing | 1.5% | 1.4% | 0.1% |
| blood orders |  |  |  |
| blood group determined | 28,365 (59%) | 7,051 (58%) | 6,580 (60%) |
| number of ordered RPCs | 0 (0, 2) | 0 (0, 2) | 0 (0, 2) |
| number of ordered FFPs | 0 (0, 2) | 0 (0, 2) | 0 (0, 2) |
| lab values |  |  |  |
| haemoglobin [g/dl] | 14.0 (12.8, 15.1) | 14.0 (12.8, 15.1) | 14.00 (12.8, 15.1) |
| unknown | 10.1% | 10.5% | 6.4% |
| haematocrit [%] | 41.0 (38.0, 43.8) | 41.1 (38.1, 43.8) | 41.0 (37.9, 43.8) |
| unknown | 10.1% | 10.5% | 6.4% |
| erythrocytes [10^12^/l] | 4.7 (4.3, 5.0) | 4.7 (4.3, 5.0) | 4.6 (4.3, 5.0) |
| unknown | 10.1% | 10.5% | 6.3% |
| MCHC [g/dl] | 34.1 (33.3, 34.8) | 34.1 (33.4, 34.8) | 34.1 (33.4, 34.8) |
| unknown | 10.2% | 10.5% | 6.4% |
| leukocytes [10^9^/l] | 6.99 (5.76, 8.52) | 6.92 (5.67, 8.46) | 6.89 (5.70, 8.44) |
| unknown | 10.1% | 10.5% | 6.3% |
| thrombocytes [10^9^/l] | 242 (203, 286) | 240 (202, 285) | 244 (206, 290) |
| unknown | 10.4% | 10.6% | 6.6% |
| Prothrombin time [%] | 102 (95, 110) | 102 (95, 110) | 117 (109, 120) |
| unknown | 10.4% | 10.7% | 6.7% |
| factor VIII.C [%] | 148 (116, 186) | 128 (101, 188) | 121 (103, 176) |
| unknown | 99.6% | 99.7% | 99.4% |
| serum potassium [mmol/l] | 4.6 (4.4, 4.9) | 4.6 (4.4, 4.9) | 4.6 (4.3, 4.8) |
| unknown | 10.2% | 10.6% | 6.3% |
| serum calcium [mmol/l] | 2.39 (2.32, 2.46) | 2.39 (2.32, 2.46) | 2.39 (2.31, 2.46) |
| unknown | 75.7% | 75.8% | 84.6% |
| serum creatinine [mg/dl] | 0.9 (0.8, 1.0) | 0.9 (0.8, 1.1) | 0.9 (0.7, 1.0) |
| unknown | 10.4% | 10.9% | 6.3% |
| GFR(CKD.EPI)[%] | 85 (69, 99) | 85 (69, 98) | 87 (71, 101) |
| unknown | 22.0% | 22.1% | 6.1% |
| BUN [mg/dl] | 15 (12, 19) | 15 (12, 19) | 15 (12, 19) |
| unknown | 62.3% | 63.4% | 64.0% |
| serum albumin [g/dl] | 4.4 (4.0, 4.7) | 4.4 (4.0, 4.6) | 4.5 (4.1, 4.7) |
| unknown | 88.5% | 88.8% | 86.6% |
| plasma cholinesterase [U/l] | 7,632 (6,333, 8,861) | 7,576 (6,257, 8,867) | 7,615 (6,333, 8,787) |
| unknown | 88.3% | 88.2% | 88.1% |
| protein [g/dl] | 7.0 (6.7, 7.4) | 7.0 (6.7, 7.4) | 6.9 (6.5, 7.3) |
| unknown | 80.0% | 80.6% | 88.1% |
| gamma-GT [U/l] | 27 (17, 48) | 27 (17, 49) | 28 (18, 52) |
| unknown | 56.6% | 57.4% | 57.1% |
| alkaline phosphatase [U/l] | 74 (61, 95) | 74 (60, 92) | 77 (61, 100) |
| unknown | 79.4% | 80% | 82.5% |
| C-reactive protein [mg/dl] | 0.2 (0.1, 0.7) | 0.2 (0.1, 0.6) | 0.2 (0.1, 0.7) |
| unknown | 40.4% | 41.2% | 39.2% |
| TSH [µIU/ml] | 1.46 (0.96, 2.15) | 1.49 (0.97, 2.17) | 1.46 (0.99, 2.15) |
| unknown | 51.9% | 52.6% | 48.3% |
| CEA [ng/ml] | 3 (2, 6) | 3 (2, 5) | 2 (1, 4) |
| unknown | 98.4% | 98.3% | 97.9% |
| year |  |  |  |
| 2014 | 2,957 (6.1%) | 726 (6.0%) | 0 (0%) |
| 2015 | 8,158 (17%) | 1,970 (16%) | 0 (0%) |
| 2016 | 9,786 (20%) | 2,466 (20%) | 0 (0%) |
| 2017 | 10,494 (22%) | 2,560 (21%) | 0 (0%) |
| 2018 | 10,484 (22%) | 2,684 (22%) | 0 (0%) |
| 2019 | 6,538 (14%) | 1,669 (14%) | 4,535 (41%) |
| 2020 | 0 (0%) | 0 (0%) | 6,491 (59%) |
| month |  |  |  |
| Jan | 4,130 (8.5%) | 1,090 (9.0%) | 1,280 (12%) |
| Feb | 4,116 (8.5%) | 977 (8.1%) | 1,172 (11%) |
| Mar | 4,199 (8.7%) | 1,052 (8.7%) | 4,039 (37%) |
| Apr | 3,981 (8.2%) | 1,011 (8.4%) | 0 (0%) |
| May | 3,919 (8.1%) | 980 (8.1%) | 0 (0%) |
| Jun | 4,167 (8.6%) | 1,066 (8.8%) | 0 (0%) |
| Jul | 4,710 (9.7%) | 1,172 (9.7%) | 0 (0%) |
| Aug | 3,794 (7.8%) | 948 (7.9%) | 0 (0%) |
| Sep | 3,765 (7.8%) | 925 (7.7%) | 1,188 (11%) |
| Oct | 4,156 (8.6%) | 993 (8.2%) | 1,241 (11%) |
| Nov | 4,143 (8.6%) | 1,027 (8.5%) | 1,181 (11%) |
| Dec | 3,337 (6.9%) | 834 (6.9%) | 925 (8.4%) |
| n (%); Median (IQR) | | | |

### ASA= American Society of Anaesthesiologists Physical Score, RPCs= red packed cells, FFPs = fresh frozen plasma units, MCHC= mean corpuscular haemoglobin concentration, factor VIII.C=antihemophilic factor A activity, GFR(CKD.EPI)= glomerular filtration rate calculated by chronic kidney disease epidemiology collaboration formula, BUN = blood urea nitrogen, gamma-GT= gamma glutamyl transferase, TSH = thyroid stimulating hormone, CEA= carcinoembryonic antigen

The parameters sex, age, number of pre-operative consults, time, day, month and year of surgery were available in each case.

### Table A2: Contribution to the prediction (importance) and frequency of the individual factors applied in the model

| **Feature** | **Importance** | **Frequency** |
| --- | --- | --- |
| red packed cells | 0.10425 | 0.04644 |
| c-reactive protein | 0.06546 | 0.03295 |
| age | 0.06476 | 0.04569 |
| American Society of Anaesthesiologists Physical Score classification | 0.04955 | 0.02022 |
| no of preop consults | 0.04610 | 0.01573 |
| fresh-frozen plasma | 0.03910 | 0.01872 |
| potassium | 0.02560 | 0.02247 |
| erythrocytes | 0.02393 | 0.01198 |
| gamma-glutamyl-transferase | 0.02164 | 0.01797 |
| prothrombin time | 0.01863 | 0.02172 |
| leukocytes | 0.01777 | 0.02022 |
| body-mass-index | 0.01679 | 0.02621 |
| albumin | 0.01639 | 0.00973 |
| cholinesterase | 0.01617 | 0.01498 |
| blood urea nitrogen | 0.01534 | 0.01722 |
| haemoglobin | 0.01259 | 0.01498 |
| thrombocytes | 0.01245 | 0.02097 |
| thyrotropin | 0.01236 | 0.01797 |
| carcinoembryonic antigen | 0.01130 | 0.00749 |
| height | 0.01105 | 0.02097 |
| alkaline phosphatase | 0.01078 | 0.00823 |
| creatinine | 0.00984 | 0.01123 |
| protein | 0.00973 | 0.01498 |
| glomerular filtration rate - modification of diet in renal disease | 0.00956 | 0.01048 |
| mean corpuscular haemoglobin | 0.00900 | 0.01348 |
| factor VIIIC | 0.00877 | 0.00898 |
| haematocrit | 0.00867 | 0.01348 |
| glomerular filtration rate chronic kidney disease epidemiology collaboration | 0.00808 | 0.01423 |
| operation and procedure code: linear stapler, open surgical, for intrathoracic or intraabdominal use | 0.00786 | 0.00674 |
| calcium | 0.00773 | 0.01498 |
| month | 0.00760 | 0.01123 |
| epidural | 0.00754 | 0.00599 |
| mallampati | 0.00747 | 0.00823 |
| weight | 0.00731 | 0.01423 |
| sodium | 0.00681 | 0.00898 |
| electrocardiogram | 0.00648 | 0.00299 |
| New York Heart Association | 0.00646 | 0.00674 |
| hour of operation time | 0.00634 | 0.00973 |
| partial thromboplastin time | 0.00562 | 0.00898 |
| ATC-Code: nasal decongestants for systemic use | 0.00559 | 0.00449 |
| point-of-care-testing | 0.00554 | 0.00449 |
| admission | 0.00527 | 0.00674 |
| bilirubin | 0.00501 | 0.00898 |
| ATC-Code: beta-blocking agents | 0.00501 | 0.00524 |
| mean corpuscular haemoglobin | 0.00482 | 0.00599 |
| international normalized ratio | 0.00472 | 0.00299 |
| prostate-specific antigen | 0.00467 | 0.00674 |
| blood type | 0.00461 | 0.00674 |
| day | 0.00456 | 0.01048 |
| total bilirubin | 0.00454 | 0.00449 |
| blood type (fully automatic) | 0.00453 | 0.01048 |
| urine leukocytes | 0.00425 | 0.00599 |
| urine bilirubin | 0.00419 | 0.00374 |
| preop consults emergency department | 0.00383 | 0.00599 |
| thyroid disease | 0.00353 | 0.00599 |
| electrocardiogram | 0.00350 | 0.00524 |
| MCV | 0.00334 | 0.00449 |
| mean corpuscular volume | 0.00328 | 0.00299 |
| preop radiological exam | 0.00318 | 0.00524 |
| ATC-Code: other drugs for obstructive airway diseases, inhalants | 0.00312 | 0.00449 |
| glutamate-oxaloacetate transaminase | 0.00311 | 0.00823 |
| lung disease | 0.00301 | 0.00299 |
| glutamate-pyruvate transaminase | 0.00281 | 0.00599 |
| ATC-Code: drugs for peptic ulcer and gastro-oesophageal reflux disease | 0.00270 | 0.00224 |
| platelet concentrates | 0.00266 | 0.00224 |
| squamous cell carcinoma antigen | 0.00264 | 0.00524 |
| former anaesthesia | 0.00260 | 0.00524 |
| activated partial thromboplastin time | 0.00260 | 0.00374 |
| operation and procedure code: vacuum therapy | 0.00259 | 0.00449 |
| bicarbonate | 0.00257 | 0.00299 |
| rhesus factor | 0.00251 | 0.00449 |
| alcohol | 0.00241 | 0.00374 |
| red packed cells | 0.00220 | 0.00074 |
| operation and procedure code: pancreatic head resection | 0.00214 | 0.00224 |
| creatine kinase | 0.00211 | 0.00449 |
| free thyroxine | 0.00211 | 0.00449 |
| year | 0.00211 | 0.00449 |
| kidney disease | 0.00208 | 0.00149 |
| ionized calcium | 0.00207 | 0.00224 |
| point-of-care-testing | 0.00204 | 0.00524 |
| specific gravity urine | 0.00204 | 0.00524 |
| cataract | 0.00202 | 0.00374 |
| anion gap | 0.00202 | 0.00374 |
| operation and procedure code | 0.00202 | 0.00374 |
| blood group control card | 0.00201 | 0.00449 |
| lipase | 0.00198 | 0.00224 |
| venous glucose | 0.00197 | 0.00524 |
| urine sediment bacteria | 0.00190 | 0.00149 |
| care assignment | 0.00185 | 0.00374 |
| ATC-Code: low-ceiling diuretics, thiazides | 0.00182 | 0.00299 |
| operation and procedure code: excision of intracerebral tumour tissue, noncerebral | 0.00177 | 0.00299 |
| compensated renal insufficiency | 0.00172 | 0.00149 |
| chest x-ray | 0.00171 | 0.00299 |
| urine protein | 0.00165 | 0.00149 |
| urine pH | 0.00164 | 0.00224 |
| alcohol occasionally | 0.00161 | 0.00299 |
| ATC-Code: hypothalamic hormones | 0.00159 | 0.00224 |
| sodium | 0.00155 | 0.00299 |
| pre-operative radiology consults | 0.00148 | 0.00299 |
| sex | 0.00147 | 0.00299 |
| no alcohol | 0.00145 | 0.00149 |
| ATC-Code: high-ceiling diuretics | 0.00144 | 0.00224 |
| chest x-ray available | 0.00141 | 0.00149 |
| sinus rhythm | 0.00137 | 0.00299 |
| von-Willebrand-factor | 0.00136 | 0.00224 |
| state after thyroidectomy | 0.00135 | 0.00074 |
| blood group determined by galileo | 0.00134 | 0.00224 |
| normotonia | 0.00133 | 0.00149 |
| creatinine | 0.00129 | 0.00074 |
| heart rate | 0.00121 | 0.00224 |
| ATC-Code: corticosteroids | 0.00119 | 0.00224 |
| total bilirubin available | 0.00113 | 0.00074 |
| bleeding time | 0.00110 | 0.00149 |
| department | 0.00109 | 0.00149 |
| prostate-specific antigen | 0.00109 | 0.00224 |
| rhesus formula | 0.00109 | 0.00224 |
| ATC-Code: anti-parathyroid agents | 0.00109 | 0.00074 |
| cancer antigen 19-9 | 0.00106 | 0.00074 |
| antibody screening test | 0.00102 | 0.00074 |
| troponin t | 0.00100 | 0.00074 |
| teeth sanitized | 0.00099 | 0.00224 |
| multiplate ADP | 0.00099 | 0.00224 |
| anaesthesiologic consults preop | 0.00098 | 0.00224 |
| urine nitrite | 0.00097 | 0.00149 |
| intraoperative vascular blood flow measurement | 0.00097 | 0.00224 |
| ATC-Code: other cold preparation | 0.00096 | 0.00149 |
| blood iron | 0.00096 | 0.00224 |
| full denture | 0.00094 | 0.00074 |
| vascular surgery consult preop | 0.00093 | 0.00149 |
| COPD | 0.00093 | 0.00074 |
| lymphocytes | 0.00091 | 0.00149 |
| cholecystectomy | 0.00087 | 0.00149 |
| blood glucose | 0.00086 | 0.00149 |
| urine ketone bodies | 0.00083 | 0.00074 |
| maxillary partial denture | 0.00082 | 0.00224 |
| urine germ cells | 0.00081 | 0.00074 |
| metabolic disorder | 0.00080 | 0.00074 |
| FFP ordered | 0.00078 | 0.00074 |
| haematocrit | 0.00078 | 0.00224 |
| ATC-Code: Angiotensin II receptor blockers | 0.00078 | 0.00074 |
| upper full denture | 0.00077 | 0.00074 |
| hypothyroidism | 0.00076 | 0.00074 |
| transitional epithelium urine | 0.00076 | 0.00074 |
| preop cardiology consult | 0.00076 | 0.00074 |
| glomerular filtration rate | 0.00075 | 0.00149 |
| electrocardiogram | 0.00074 | 0.00149 |
| serum laboratory taken | 0.00073 | 0.00149 |
| operation and procedure code: relaparotomy | 0.00073 | 0.00074 |
| rhesus formula determined by galileo | 0.00071 | 0.00149 |
| ATC-Code: Beta-blocking agents, other combinations | 0.00068 | 0.00149 |
| general anaesthesia planned | 0.00067 | 0.00149 |
| rhesus factor quick determination | 0.00067 | 0.00149 |
| hyperlipidaemia/hypercholesterinaemia | 0.00065 | 0.00149 |
| O2 saturation | 0.00065 | 0.00149 |
| fraction of carboxyhaemoglobin | 0.00062 | 0.00149 |
| chest x-ray normal finding | 0.00062 | 0.00074 |
| lactate dehydrogenase | 0.00062 | 0.00224 |
| preop consult nuclear medicine | 0.00061 | 0.00074 |
| von-Willebrand-factor activity | 0.00061 | 0.00074 |
| operation and procedure code: retroperitoneal lymphadenectomy | 0.00059 | 0.00149 |
| sodium POCT | 0.00058 | 0.00074 |
| main diagnosis available | 0.00056 | 0.00074 |
| neurologic disorder | 0.00053 | 0.00074 |
| O2 saturation | 0.00052 | 0.00149 |
| skeletal disease | 0.00052 | 0.00074 |
| consult dialysis/nephrology | 0.00052 | 0.00074 |
| asthma | 0.00047 | 0.00074 |
| operation and procedure code: operation on the blood vessels: femoral artery | 0.00047 | 0.00074 |
| number of OPS codes starting with 1 | 0.00046 | 0.00074 |
| half saturation HbO2 | 0.00044 | 0.00074 |
| penicillin allergy | 0.00043 | 0.00074 |
| weekday | 0.00042 | 0.00149 |
| ATC-Code: anaesthetics | 0.00039 | 0.00074 |
| ATC-Code: selective calcium channel blockers with mainly vascular effects | 0.00039 | 0.00074 |
| oxyhaemoglobin fraction | 0.00037 | 0.00074 |
| phosphate | 0.00037 | 0.00074 |
| point-of-care-testing | 0.00036 | 0.00074 |
| eye diseases | 0.00035 | 0.00074 |
| operation and procedure code | 0.00035 | 0.00224 |
| fibrinogen | 0.00034 | 0.00074 |
| fully automated blood group determination | 0.00034 | 0.00074 |
| lactate | 0.00034 | 0.00074 |
| monocytes | 0.00032 | 0.00074 |
| neutrophiles | 0.00031 | 0.00074 |
| eosinophiles | 0.00030 | 0.00074 |
| analgosedation planned | 0.00029 | 0.00074 |
| base excess | 0.00028 | 0.00074 |
| ATC-Code: adrenergics, inhalants | 0.00028 | 0.00074 |
| blood pH | 0.00026 | 0.00074 |
| non-smoker | 0.00026 | 0.00074 |
| HbO2 capacity | 0.00024 | 0.00074 |
| urine sediment urate | 0.00024 | 0.00074 |
| standard HCO3 | 0.00023 | 0.00074 |
| methaemoglobin fraction | 0.00023 | 0.00074 |
| deoxyhaemoglobin fraction | 0.00021 | 0.00074 |
| operation and procedure code: access to lumbar spine, more than 2 segments | 0.00021 | 0.00074 |
| uric acid | 0.00021 | 0.00074 |
| ATC-Code: hormone antagonists and related agents | 0.00021 | 0.00074 |
| x-ray ordered but not done | 0.00019 | 0.00074 |
| ATC-Code: other alimentary tract and metabolism products | 0.00019 | 0.00074 |
| allergies | 0.00013 | 0.00074 |

### Factor VIII.C=antihaemophilic factor A activity, ATC code = anatomic therapeutic chemical code, MCV=mean corpuscular volume, multiplate ADP = ADP-induced platelet activation for sensitive detection of the efficacy of clopidogrel, prasugrel, and other ADP receptor antagonists, COPD=chronic obstructive pulmonary disease, FFP= fresh frozen plasma, POCT=point-of-care testing

### Table A3: Calibration metrics

|  | Original | Platt | Isotonic |
| --- | --- | --- | --- |
| Brier Score | 0.005329215 | 0.004640364 | 0.004280265 |
| ICI | 0.009725718 | 0.01828012 | 0.01631023 |
| Spiegelhalter_z (p.-value) | <0.0001 | 0.1733476 | 0.0687834 |
| Hosmer Lemeshow (p-value) | <0.0001 | <0.0001 | 0.4040688 |

ICI= integrated calibration index

### Figure A4: Calibration plots

Calibration curves of the original (unclibrated) model and the models calibrated by means of isotonic regression and Platt scaling. Isotonic calibration shows the best fit.

Figure A5: Prediction metrics

Negative predictive value(NPV), precision(=positive predictive value, PPV), F1 score and recall (=sensitivity) depending on the calculated probability of the XGBoost model.

### R-script for model creation

####
# Explicable Mortality ###

#####
# load libraries ####

install.packages("tidymodels") install.packages("gtsummary") install.packages("skimr") install.packages("purrr") install.packages("xgboost") install.packages("miceadds") install.packages("tidyr") install.packages("rBayesianOptimization") install.packages("mlrMBO") install.packages("DiceKriging") install.packages("rgenoud") install.packages("pROC")

library(tidymodels)
library(gtsummary)
library(tidyr)
library(dplyr)
library(xgboost)
library(tidyverse) # for data wrangling library(rBayesianOptimization) # to create cv folds and for bayesian optimisation

library(mlrMBO) # for bayesian optimisation require(skimr) # for summarising databases library(purrr) # to evaluate the loglikelihood of each parameter set in the random grid search require("DiceKriging") # mlrmbo requires this require("rgenoud") # mlrmbo requires this library(pROC)

library(PRROC)

###
# load Data ###

risk <- miceadds::load.data(file="Risiko_bis_Okt_2021 numerisch.RData",type="RData",path="../Bewegungsd aten/BewegungsdatenWS/Mortality/")

table(risk$verstorben)

####
# Create trainig/test/validation frame ###

risk$my <- paste(risk$month,risk$year,sep="_") mort_df <- risk %>% filter(my %in% c("6_2014",

"7_2014", "8_2014", "9_2014", "10_2014", "11_2014", "12_2014", "1_2015", "2_2015", "3_2015", "4_2015", "5_2015", "6_2015", "7_2015", "8_2015", "9_2015", "10_2015", "11_2015", "12_2015", "1_2016", "2_2016",

"3_2016", "4_2016", "5_2016", "6_2016", "7_2016", "8_2016", "9_2016", "10_2016", "11_2016", "12_2016", "1_2017", "2_2017", "3_2017", "4_2017", "5_2017", "6_2017", "7_2017", "8_2017", "9_2017", "10_2017", "11_2017", "12_2017", "1_2018", "2_2018", "3_2018", "4_2018", "5_2018", "6_2018", "7_2018", "8_2018", "9_2018", "10_2018", "11_2018", "12_2018", "1_2019", "2_2019", "3_2019", "4_2019", "5_2019", "6_2019",

dim(mort_df)

##############################

##############################

##############################

##############################

######

##. Elective patients over 17 ############################## ############################## ############################## ############################## ######

mort_df_electiv <- mort_df %>% filter(FB_PMP_OPPRIORITY==2) %>% select(- c(Patient,LfdNr, PDMS_OP_ID, PDMS_KLIN_AUFTRAG, SAP_KLIN_AUFTRAG, PDMS_PAT_ID, SAP_PAT_ID, PDMS_FALLNUMMER, Intervention_ID)) mort_df_electiv18 <- mort_df_electiv %>% filter(Alter>17)

mort_df_electiv18_tt <- mort_df_electiv18 %>% filter(my %in% c("6_2014",

"7_2019", "8_2019", "9_2019", "10_2019", "11_2019", "12_2019", "1_2020", "2_2020", "3_2020"))

"7_2014", "8_2014", "9_2014", "10_2014", "11_2014", "12_2014", "1_2015", "2_2015", "3_2015", "4_2015", "5_2015", "6_2015", "7_2015", "8_2015", "9_2015", "10_2015", "11_2015", "12_2015", "1_2016", "2_2016",

"3_2016", "4_2016", "5_2016", "6_2016", "7_2016", "8_2016", "9_2016", "10_2016", "11_2016", "12_2016", "1_2017", "2_2017", "3_2017", "4_2017", "5_2017", "6_2017", "7_2017", "8_2017", "9_2017", "10_2017",

"11_2017", "12_2017", "1_2018", "2_2018", "3_2018", "4_2018", "5_2018", "6_2018", "7_2018", "8_2018", "9_2018", "10_2018", "11_2018", "12_2018", "1_2019", "2_2019", "3_2019", "4_2019", "5_2019", "6_2019",

"7_2019", "8_2019"))

set.seed(1234)

xgboost_data_split <- initial_split(mort_df_electiv18_tt, strata = verstorben, prop=.8)
train_em <- training(xgboost_data_split) test_em <- testing(xgboost_data_split)

val_em <- mort_df_electiv18 %>% filter(!my %in%

c("6_2014",

"7_2014", "8_2014", "9_2014", "10_2014", "11_2014", "12_2014", "1_2015", "2_2015", "3_2015", "4_2015", "5_2015", "6_2015", "7_2015", "8_2015", "9_2015", "10_2015", "11_2015",

"12_2015", "1_2016", "2_2016", "3_2016", "4_2016", "5_2016", "6_2016", "7_2016", "8_2016", "9_2016", "10_2016", "11_2016", "12_2016", "1_2017", "2_2017", "3_2017", "4_2017", "5_2017", "6_2017", "7_2017", "8_2017", "9_2017", "10_2017", "11_2017", "12_2017", "1_2018", "2_2018", "3_2018", "4_2018", "5_2018", "6_2018", "7_2018", "8_2018", "9_2018", "10_2018", "11_2018", "12_2018", "1_2019", "2_2019", "3_2019",

dim(train_em) dim(test_em) dim(val_em)

names(train_em)[537]

train_em_17Fall <- train_em %>% filter(Alter>17) %>% select(Fall)
test_em_17Fall <- test_em %>% filter(Alter>17) %>% select(Fall)

val_em_17Fall <- val_em %>% filter(Alter>17) %>% select(Fall)

train_em<- train_em%>% select(-Fall) test_em <- test_em %>% select(-Fall) val_em <- val_em %>% select(-Fall)

train_em <- train_em %>% select(-my) test_em <- test_em %>% select(-my) val_em <- val_em %>% select(-my)

"4_2019", "5_2019", "6_2019", "7_2019", "8_2019"))

###
# Clean up data ###

X_train <- as.matrix(train_em %>% select(- intersect(names(train_em),c("Fall","IntensivDanach","v erstorben","Thrombos","Quick","Kalium","my","Patient ","LfdNr","Fall","PDMS_OP_ID"))))

val_em_mat <- val_em %>% select(- intersect(names(val_em ),c("Fall","IntensivDanach","verstorben","Thrombos","Q uick","Kalium","my","Patient","LfdNr","Fall","PDMS_ OP_ID","Billi","Kreatinin","Hkt","Natrium")))

xgtrain_em <- xgb.DMatrix(as.matrix(train_em %>% select(- intersect(names(train_em),c("Fall","IntensivDanach","v erstorben","Thrombos","Quick","Kalium","my","Patient ","LfdNr","Fall","PDMS_OP_ID","Billi","Kreatinin","H kt","Natrium")))), label = train_em$verstorben) xgtest_em <- xgb.DMatrix(as.matrix(test_em

%>% select(-intersect(names(test_em ),c("Fall","IntensivDanach","verstorben","Thrombos","Q uick","Kalium","my","Patient","LfdNr","Fall","PDMS_ OP_ID","Billi","Kreatinin","Hkt","Natrium")))), label = test_em$verstorben)

xvalidation_em <- xgb.DMatrix(as.matrix(val_em_mat), label = val_em$verstorben)

X_val <- as.matrix(val_em_mat %>% select(- intersect(names(val_em_mat),c("Fall","IntensivDanach ","verstorben","Thrombos","Quick","Kalium","my","Pat ient","LfdNr","Fall","PDMS_OP_ID","PDMS_OP_ID" ,"Billi","Kreatinin","Hkt","Natrium"))))

############################## #
# Hyperparameter tuning
# ##############################

###### Bayes ##### switch_generate_interim_data <- FALSE

cv_folds = KFold(train_em$verstorben, nfolds= 3,

stratified = TRUE, seed= 0)

obj.fun <- smoof::makeSingleObjectiveFunction( name = "xgb_cv_bayes",
fn = function(x){

set.seed(12345)
cv <- xgb.cv(params = list(

#booster
eta
max_depth
min_child_weight = x["min_child_weight"], gamma = x["gamma"],

subsample = x["subsample"], colsample_bytree = x["colsample_bytree"], scale_pos_weight = x["scale_pos_weight"], eval_metric = "aucpr"),
data = xgtrain_em,
nround = 1000,
folds= cv_folds,
prediction = FALSE,

= "gbtree", = x["eta"],

= x["max_depth"],

showsd = TRUE, early_stopping_rounds = 50, verbose = 1,
print_every_n = 20)

cv$evaluation_log[, max(test_aucpr_mean)] },

par.set = makeParamSet( makeNumericParam("eta",

upper = 1), makeNumericParam("gamma",

upper = 15), makeIntegerParam("max_depth",

lower = 0, lower = 0, lower= 2L,

upper = 30L), makeIntegerParam("min_child_weight", lower= 0,

upper = 25),
makeNumericParam("subsample", lower = 0,

upper = 1),
makeNumericParam("colsample_bytree", lower = 0,

upper = 1),
makeNumericParam("scale_pos_weight", lower = 0,

upper = 20) ),

minimize = FALSE )

control = makeMBOControl(on.surrogate.error="warn",save.on.di sk.at=1:120)
control = setMBOControlTermination(control, iters = 100)

run = mbo(fun = obj.fun,
# design = final_design, control = control,
show.info = TRUE)

###
# Learn model ##
# Watchlist

wl_em <- list(train=xgtrain_em,test=xgtest_em) # # eXtreme Gradient Boosting Model
Modell <- xgb.train("objective" = "binary:logistic",

eval_metric = "aucpr", data = xgtrain_em, nrounds = 10000, watchlist = wl_em,

eta = run$x$eta,
max.depth = run$x$max_depth, gamma = run$x$gamma, subsample = run$x$subsample, colsample_bytree =

run$x$colsample_bytree, scale_pos_weight =

run$x$scale_pos_weight, missing = NA,

seed = 332, early_stopping_rounds = 500, maximize = TRUE, nthread=20,
print_every_n = 10, min_child_weight =

run$x$min_child_weight)

imp_Model <- xgb.importance(feature_names =colnames(xgtrain_em),model=Modell) xgb.plot.importance(imp_Model,top_n=20)

imp_Model <- miceadds::load.data(file="imp_Model17.RData",type=" RData") write.csv2(imp_Model,file="imp_Model17.csv",fileEnco ding = "mac")
